# Bis[tris­(3,5-dimethyl-1*H*-pyrazol-1-yl-κ*N* ^2^)meth­yl]sodium trifluoro­methane­sulfonate

**DOI:** 10.1107/S1600536812028413

**Published:** 2012-06-30

**Authors:** Ganna Lyubartseva, Sean Parkin, Uma Prasad Mallik

**Affiliations:** a Department of Chemistry and Physics, Southern Arkansas University, Magnolia, AR 71753, USA; b Department of Chemistry, University of Kentucky, Lexington, KY 40506, USA

## Abstract

In the title salt, [Na(C_16_H_22_N_6_)_2_]CF_3_SO_3_, the Na^+^ cation is coordinated by six N atoms from two tridentate tris­(3,5-dimethyl­pyrazol-1-yl)methane ligands in a distorted octa­hedral geometry. The Na—N distances range from 2.427 (3) to 2.507 (3) Å, intra-ligand N—Na—N angles range from 74.71 (8) to 79.31 (9)°, and adjacent inter-ligand N—Na—N angles range between 100.42 (9) and 104.97 (9)°. The structure is twinned by inversion [occupancy factors = 0.50 (9)] and the trifluoro­methane­sulfonate anion is disordered, with two end-over-end orientations of unequal occupancy [0.781 (3) and 0.219 (3)].

## Related literature

For ligand synthesis details, see: Reger *et al.* (2000 ▶). For structural, spectroscopic and angular overlap studies of tris(pyrazol-1-yl)methane complexes, see: Astley *et al.* (1993 ▶). For details of the refinement strategy for twinned and disordered structures, see: Parkin (2000 ▶); Spek (2009 ▶).
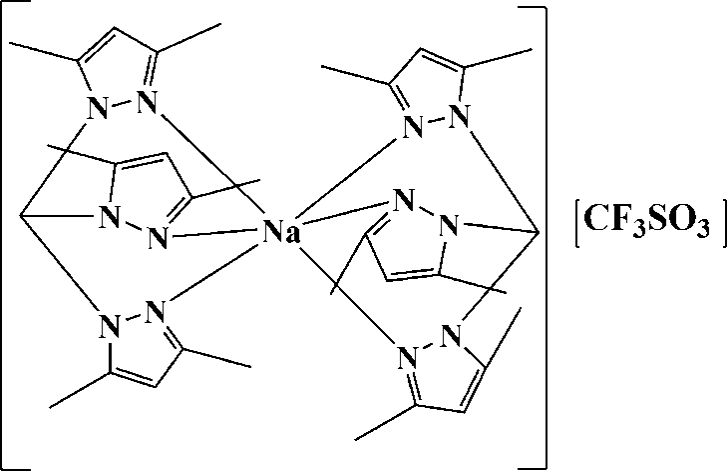


## Experimental

### 

#### Crystal data

[Na(C_16_H_22_N_6_)_2_]CF_3_O_3_S
*M* *_r_* = 768.85
Monoclinic, 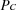

*a* = 9.0579 (1) Å
*b* = 12.5733 (1) Å
*c* = 16.4386 (2) Å
β = 90.5917 (4)°
*V* = 1872.05 (3) Å^3^
*Z* = 2
Mo *K*α radiation
μ = 0.17 mm^−1^
*T* = 90 K
0.22 × 0.18 × 0.12 mm

#### Data collection

Nonius KappaCCD diffractometer
Absorption correction: multi-scan [*SCALEPACK* (Otwinowski & Minor, 1997 ▶) and *XABS2* (Parkin *et al.* 1995 ▶)] *T*_min_ = 0.965, *T*_max_ = 0.981
47745 measured reflections
8385 independent reflections
7036 reflections with *I* > 2σ(*I*)
*R*_int_ = 0.043

#### Refinement

*R*[*F* ^2^ > 2σ(*F* ^2^)] = 0.041
*wR*(*F* ^2^) = 0.112
*S* = 1.09
8385 reflections
516 parameters
59 restraints
H-atom parameters constrained
Δρ_max_ = 0.26 e Å^−3^
Δρ_min_ = −0.27 e Å^−3^

### 

Data collection: *COLLECT* (Nonius, 1998 ▶); cell refinement: *SCALEPACK* (Otwinowski & Minor, 1997 ▶); data reduction: *DENZO-SMN* (Otwinowski & Minor, 1997 ▶); program(s) used to solve structure: *SHELXS97* (Sheldrick, 2008 ▶); program(s) used to refine structure: *SHELXL97* (Sheldrick, 2008 ▶); molecular graphics: *XP* in *SHELXTL* (Sheldrick, 2008 ▶); software used to prepare material for publication: *SHELXL97* and local procedures.

## Supplementary Material

Crystal structure: contains datablock(s) global, I. DOI: 10.1107/S1600536812028413/tk5119sup1.cif

Structure factors: contains datablock(s) I. DOI: 10.1107/S1600536812028413/tk5119Isup2.hkl

Additional supplementary materials:  crystallographic information; 3D view; checkCIF report

## Figures and Tables

**Fig. 1.**
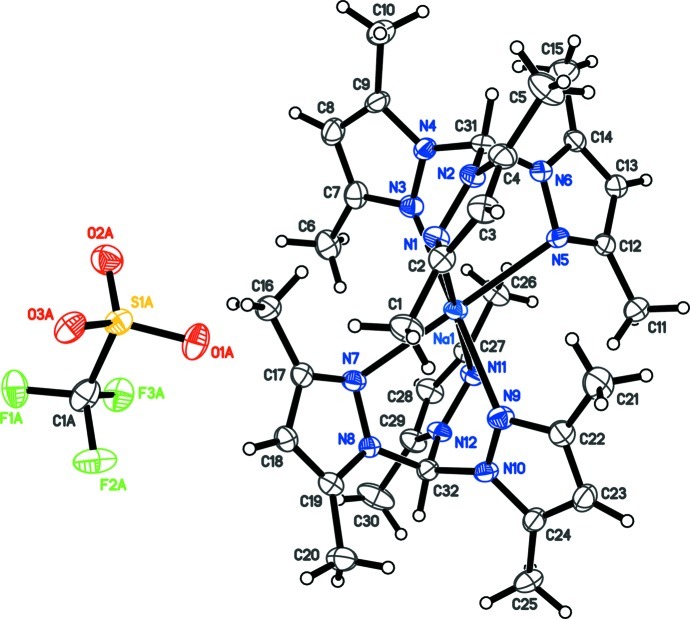
A view of the title compound with displacement ellipsoids drawn at the 50% probability level.

## supplementary crystallographic information

### Comment

In an attempt to prepare mononuclear [(tpm^Me2^)Ni^II^*L*_3_]^-1^, where tpm^Me2^ is tris(3,5-dimethylpyrazol-1-yl)methane, a symmetrical tridentate neutral nitrogen donor ligand (Astley *et al.*, 1993), and *L* is CN^-^, a mononegative N-donor anion, we isolated a minor product [(C_16_H_22_N_6_)_2_Na][CF_3_SO_3_] as pale yellow crystals. In the crystal, the sodium ion is coordinated by six N atoms from the two tridentate tpm^Me2^ ligands (average Na—N distance = 2.47 Å) in a distorted octahedral geometry. The average N—Na—N angle between adjacent pyrazole-ring-coordinated N atoms is 77.0° for the six acute angles and 103.0° for the six obtuse angles. The trifluoromethanesulfonate anion is disordered end-over-end, with the two orientations having unequal occupancy factors. The structure is twinned by inversion [occupancy factors 0.50 (9)], but the unequal occupancy of the disorder components effectively ensures the lack of inversion symmetry observed for this structure.

### Experimental

Tris(pyrazolyl)methane was synthesized according to the previously published procedure of Reger *et al.* (2000). Nickel trifluoromethanesulfonate and sodium cyanide were used as received. Ni(OTf)_2_ (358 mg, 1 mmol) was dissolved in 40 ml methanol. Tris(3,5-dimethylpyrazol-1-yl)methane (298 mg, 1 mmol) was dissolved in 25 ml methanol. The ligand solution was added dropwise to the metal solution with moderate stirring. Once the addition was complete, sodium cyanide (147 mg, 3 mmol) dissolved in 20 ml (1:1) mixture of water and methanol added. The clear solution gave precipitates that were filtered. From the clear filtrate, pale yellow crystals were obtained after 2 weeks (59 mg, 7.67% yield). IR (cm^-1^): 2924, 1564, 1447, 1414, 1384, 1366, 1319, 1262, 1133, 1031, 974, 888, 860, 809, 789, 750, 705, 635, 570, 517, 478.

### Refinement

H atoms were found in difference Fourier maps and subsequently placed in idealized positions with constrained distances of 0.98 Å (RCH_3_), 1.00 Å (*R*_3_CH), and 0.95 Å (C_sp2_H), and with *U*_iso_(H) values set to either 1.2*U*_eq_ or 1.5*U*_eq_ (RCH_3_) of the attached atom.

To ensure satisfactory refinement of disordered parts of the structure, a combination of constraints and restraints were used. The *SHELXL97* constraints EXYZ and EADP were used to make the geometry and displacement parameters of closely proximate disordered atoms equal. The *SHELXL97* restraint command DELU was also used to ensure similar displacement parameters for closely proximate, chemically similar groups.

The structure was initially solved and partly refined using space group *P*2_1_/c. That model seemed to refine quite well but there were a number of indications that something was not quite right. In particular, the difference map was not as flat as would be expected for this kind of structure, and the weighting scheme took on unusual values. The centrosymmetric model forced the disorder of the trifluoromethanesulfonate anion to be exactly 50:50. The non-centrosymmetric model in space group *P*c presented here refines better, with the trifluoromethanesulfonate anion disorder occupancies refining to 0.781 (3) and 0.219 (3) for the major and minor components respectively. This lower symmetry model is twinned by inversion, with essentially equal occupancy parts [0.50 (9)]. Additional checks using an *R*-tensor (Parkin, 2000) and *PLATON* (Spek, 2009) confirmed that the model using space group *P*c is superior to that using *P*2_1_/c.

### Crystal data

**Table:**

[Na(C_16_H_22_N_6_)_2_]CF_3_O_3_S	*F*(000) = 808
*M**_r_* = 768.85	*D*_x_ = 1.364 Mg m^−^^3^
Monoclinic, *P**c*	Mo *K*α radiation, λ = 0.71073 Å
Hall symbol: P -2yc	Cell parameters from 4496 reflections
*a* = 9.0579 (1) Å	θ = 1.0–27.5°
*b* = 12.5733 (1) Å	µ = 0.17 mm^−^^1^
*c* = 16.4386 (2) Å	*T* = 90 K
β = 90.5917 (4)°	Block, pale yellow
*V* = 1872.05 (3) Å^3^	0.22 × 0.18 × 0.12 mm
*Z* = 2	

### Data collection

**Table:**

Nonius KappaCCD diffractometer	8385 independent reflections
Radiation source: fine-focus sealed tube	7036 reflections with *I* > 2σ(*I*)
Graphite monochromator	*R*_int_ = 0.043
Detector resolution: 9.1 pixels mm^-1^	θ_max_ = 27.5°, θ_min_ = 1.6°
ω scans at fixed χ = 55°	*h* = −11→11
Absorption correction: multi-scan [*SCALEPACK* (Otwinowski & Minor, 1997) and *XABS2* (Parkin *et al.* 1995)]	*k* = −16→16
*T*_min_ = 0.965, *T*_max_ = 0.981	*l* = −21→21
47745 measured reflections	

### Refinement

**Table:**

Refinement on *F*^2^	Secondary atom site location: difference Fourier map
Least-squares matrix: full	Hydrogen site location: inferred from neighbouring sites
*R*[*F*^2^ > 2σ(*F*^2^)] = 0.041	H-atom parameters constrained
*wR*(*F*^2^) = 0.112	*w* = 1/[σ^2^(*F*_o_^2^) + (0.0596*P*)^2^ + 0.4716*P*] where *P* = (*F*_o_^2^ + 2*F*_c_^2^)/3
*S* = 1.09	(Δ/σ)_max_ < 0.001
8385 reflections	Δρ_max_ = 0.26 e Å^−^^3^
516 parameters	Δρ_min_ = −0.27 e Å^−^^3^
59 restraints	Absolute structure: nd
Primary atom site location: structure-invariant direct methods	Flack parameter: 0.50 (9)

### Special details

**Table:**

Geometry. All e.s.d.'s (except the e.s.d. in the dihedral angle between two l.s. planes) are estimated using the full covariance matrix. The cell e.s.d.'s are taken into account individually in the estimation of e.s.d.'s in distances, angles and torsion angles; correlations between e.s.d.'s in cell parameters are only used when they are defined by crystal symmetry. An approximate (isotropic) treatment of cell e.s.d.'s is used for estimating e.s.d.'s involving l.s. planes.
Refinement. Refinement of *F*^2^ against all reflections. The weighted *R*-value *wR* and goodness of fit *S* are based on *F*^2^. Conventional *R*-values *R* are based on *F*, with *F* set to zero for negative *F*^2^. The threshold expression of *F*^2^ > 2σ(*F*^2^) is used only for calculating *R*-factors(gt) *etc*. and is not relevant to the choice of reflections for refinement. *R*-values based on *F*^2^ are statistically about twice as large as those based on *F*, and *R*-values based on ALL data will be even larger.

### Fractional atomic coordinates and isotropic or equivalent isotropic displacement parameters (Å^2^)

**Table:**

	*x*	*y*	*z*	*U*_iso_*/*U*_eq_	Occ. (<1)
Na1	1.00452 (13)	0.75027 (11)	0.49911 (7)	0.02050 (15)	
N1	0.9369 (2)	0.60685 (18)	0.40544 (14)	0.0221 (5)	
N2	0.8555 (3)	0.63949 (17)	0.33939 (14)	0.0209 (5)	
N3	1.1108 (3)	0.80132 (17)	0.36741 (14)	0.0228 (5)	
N4	1.0369 (2)	0.76747 (18)	0.29950 (14)	0.0217 (5)	
N5	0.7809 (2)	0.82234 (17)	0.42687 (13)	0.0208 (5)	
N6	0.8017 (3)	0.82584 (17)	0.34415 (13)	0.0203 (5)	
N7	1.2291 (3)	0.67937 (17)	0.57030 (13)	0.0220 (5)	
N8	1.2082 (3)	0.67406 (17)	0.65249 (13)	0.0200 (5)	
N9	0.8992 (3)	0.69786 (18)	0.63046 (14)	0.0249 (5)	
N10	0.9732 (3)	0.73381 (19)	0.69852 (13)	0.0217 (5)	
N11	1.0735 (3)	0.89308 (18)	0.59129 (14)	0.0226 (5)	
N12	1.1548 (3)	0.86073 (18)	0.65807 (14)	0.0222 (5)	
C1	0.9347 (4)	0.4552 (2)	0.49948 (19)	0.0312 (7)	
H1A	1.0388	0.4385	0.4901	0.047*	
H1B	0.8790	0.3890	0.5061	0.047*	
H1C	0.9261	0.4984	0.5488	0.047*	
C2	0.8737 (3)	0.5159 (2)	0.42806 (17)	0.0238 (6)	
C3	0.7522 (4)	0.4915 (2)	0.37739 (19)	0.0287 (6)	
H3	0.6896	0.4312	0.3813	0.034*	
C4	0.7422 (3)	0.5713 (2)	0.32169 (18)	0.0258 (7)	
C5	0.6373 (4)	0.5885 (3)	0.2528 (2)	0.0394 (8)	
H5A	0.5680	0.5288	0.2496	0.059*	
H5B	0.6922	0.5934	0.2019	0.059*	
H5C	0.5825	0.6547	0.2615	0.059*	
C6	1.3664 (4)	0.8453 (2)	0.4042 (2)	0.0334 (7)	
H6A	1.3784	0.9226	0.4003	0.050*	
H6B	1.4603	0.8104	0.3919	0.050*	
H6C	1.3363	0.8265	0.4594	0.050*	
C7	1.2513 (3)	0.8096 (2)	0.34495 (18)	0.0270 (6)	
C8	1.2667 (3)	0.7796 (2)	0.26251 (19)	0.0304 (7)	
H8	1.3557	0.7780	0.2324	0.036*	
C9	1.1288 (3)	0.7535 (2)	0.23452 (15)	0.0258 (6)	
C10	1.0736 (4)	0.7178 (3)	0.15349 (18)	0.0346 (7)	
H10A	1.0299	0.6468	0.1584	0.052*	
H10B	1.1559	0.7152	0.1153	0.052*	
H10C	0.9987	0.7678	0.1334	0.052*	
C11	0.6788 (3)	0.9413 (2)	0.52994 (17)	0.0282 (6)	
H11A	0.5710	0.9446	0.5333	0.042*	
H11B	0.7211	1.0099	0.5461	0.042*	
H11C	0.7161	0.8855	0.5664	0.042*	
C12	0.7221 (3)	0.9165 (2)	0.44440 (17)	0.0222 (6)	
C13	0.7085 (3)	0.9811 (2)	0.37506 (17)	0.0238 (6)	
H13	0.6698	1.0512	0.3723	0.029*	
C14	0.7630 (3)	0.9216 (2)	0.31187 (18)	0.0216 (6)	
C15	0.7818 (4)	0.9479 (3)	0.22412 (18)	0.0361 (8)	
H15A	0.8861	0.9409	0.2097	0.054*	
H15B	0.7493	1.0212	0.2141	0.054*	
H15C	0.7225	0.8991	0.1908	0.054*	
C16	1.3277 (3)	0.5600 (2)	0.46573 (17)	0.0278 (6)	
H16A	1.2628	0.5989	0.4281	0.042*	
H16B	1.3179	0.4835	0.4560	0.042*	
H16C	1.4303	0.5817	0.4570	0.042*	
C17	1.2853 (3)	0.5845 (2)	0.55116 (17)	0.0212 (6)	
C18	1.2962 (3)	0.5187 (2)	0.62044 (17)	0.0230 (6)	
H18	1.3322	0.4477	0.6222	0.028*	
C19	1.2452 (3)	0.5763 (2)	0.68423 (18)	0.0234 (6)	
C20	1.2240 (4)	0.5492 (3)	0.77164 (17)	0.0317 (7)	
H20A	1.2604	0.4770	0.7821	0.048*	
H20B	1.1187	0.5530	0.7846	0.048*	
H20C	1.2789	0.5998	0.8057	0.048*	
C21	0.6434 (4)	0.6550 (3)	0.5950 (2)	0.0360 (7)	
H21A	0.6856	0.6493	0.5405	0.054*	
H21B	0.5618	0.7062	0.5939	0.054*	
H21C	0.6064	0.5853	0.6120	0.054*	
C22	0.7596 (3)	0.6920 (2)	0.65368 (17)	0.0258 (6)	
C23	0.7438 (3)	0.7229 (2)	0.73555 (19)	0.0300 (7)	
H23	0.6550	0.7248	0.7658	0.036*	
C24	0.8825 (3)	0.7497 (2)	0.76276 (17)	0.0260 (6)	
C25	0.9384 (4)	0.7873 (3)	0.84351 (18)	0.0363 (7)	
H25A	0.8585	0.7842	0.8832	0.054*	
H25B	0.9734	0.8608	0.8387	0.054*	
H25C	1.0200	0.7417	0.8617	0.054*	
C26	1.0768 (4)	1.0433 (2)	0.49665 (19)	0.0313 (7)	
H26A	1.0121	0.9969	0.4642	0.047*	
H26B	1.1586	1.0681	0.4630	0.047*	
H26C	1.0203	1.1045	0.5160	0.047*	
C27	1.1367 (3)	0.9827 (2)	0.56771 (17)	0.0233 (6)	
C28	1.2602 (4)	1.0071 (2)	0.61699 (19)	0.0288 (7)	
H28	1.3242	1.0665	0.6117	0.035*	
C29	1.2696 (3)	0.9280 (2)	0.67423 (19)	0.0274 (7)	
C30	1.3762 (4)	0.9100 (3)	0.7431 (2)	0.0443 (9)	
H30A	1.3229	0.9100	0.7947	0.066*	
H30B	1.4501	0.9670	0.7438	0.066*	
H30C	1.4255	0.8413	0.7359	0.066*	
C31	0.8823 (3)	0.7424 (2)	0.30292 (15)	0.0220 (5)	
H31	0.8443	0.7393	0.2456	0.026*	
C32	1.1295 (3)	0.7578 (2)	0.69449 (15)	0.0206 (5)	
H32	1.1686	0.7606	0.7516	0.025*	
S1A	1.42401 (13)	0.23792 (11)	0.45046 (7)	0.0236 (3)	0.781 (3)
O1A	1.2874 (5)	0.2810 (6)	0.4826 (4)	0.0341 (8)	0.781 (3)
O2A	1.4806 (6)	0.2936 (4)	0.3817 (3)	0.0346 (8)	0.781 (3)
O3A	1.4309 (10)	0.1217 (5)	0.4462 (4)	0.0285 (9)	0.781 (3)
C1A	1.5571 (9)	0.2680 (8)	0.5306 (5)	0.0315 (13)	0.781 (3)
F1A	1.6911 (4)	0.2312 (5)	0.5142 (4)	0.0366 (7)	0.781 (3)
F2A	1.5172 (8)	0.2272 (6)	0.6019 (3)	0.0419 (8)	0.781 (3)
F3A	1.5720 (9)	0.3734 (4)	0.5410 (5)	0.0337 (9)	0.781 (3)
S1A'	1.5809 (9)	0.2649 (8)	0.5447 (4)	0.0236 (3)	0.219 (3)
O1A'	1.719 (2)	0.218 (2)	0.5171 (19)	0.0366 (7)	0.219 (3)
O2A'	1.521 (4)	0.216 (3)	0.6152 (15)	0.0419 (8)	0.219 (3)
O3A'	1.583 (5)	0.3808 (16)	0.552 (2)	0.0337 (9)	0.219 (3)
C1A'	1.452 (2)	0.2410 (17)	0.4626 (11)	0.0315 (13)	0.219 (3)
F1A'	1.3170 (18)	0.2813 (19)	0.4720 (14)	0.0341 (8)	0.219 (3)
F2A'	1.504 (2)	0.2688 (13)	0.3871 (10)	0.0346 (8)	0.219 (3)
F3A'	1.426 (3)	0.1342 (15)	0.4598 (15)	0.0285 (9)	0.219 (3)

### Atomic displacement parameters (Å^2^)

**Table:**

	*U*^11^	*U*^22^	*U*^33^	*U*^12^	*U*^13^	*U*^23^
Na1	0.0208 (3)	0.0242 (3)	0.0164 (3)	−0.0025 (2)	−0.0023 (2)	−0.0001 (2)
N1	0.0231 (12)	0.0239 (12)	0.0193 (11)	−0.0016 (9)	−0.0032 (9)	0.0026 (9)
N2	0.0219 (12)	0.0209 (11)	0.0199 (11)	−0.0027 (9)	−0.0027 (9)	0.0008 (9)
N3	0.0234 (12)	0.0238 (11)	0.0212 (11)	−0.0023 (9)	−0.0021 (9)	−0.0015 (9)
N4	0.0208 (11)	0.0248 (12)	0.0197 (10)	−0.0005 (9)	0.0028 (8)	−0.0005 (9)
N5	0.0202 (12)	0.0267 (12)	0.0156 (11)	−0.0004 (9)	0.0008 (9)	−0.0015 (9)
N6	0.0213 (12)	0.0221 (11)	0.0174 (11)	0.0014 (9)	−0.0002 (9)	−0.0004 (9)
N7	0.0237 (13)	0.0250 (12)	0.0173 (11)	−0.0003 (9)	−0.0023 (10)	0.0000 (9)
N8	0.0215 (12)	0.0207 (11)	0.0179 (11)	0.0022 (9)	0.0001 (9)	−0.0001 (9)
N9	0.0249 (13)	0.0285 (12)	0.0213 (11)	−0.0047 (10)	0.0004 (10)	0.0008 (9)
N10	0.0230 (11)	0.0263 (12)	0.0158 (10)	−0.0026 (10)	0.0004 (8)	−0.0007 (9)
N11	0.0248 (12)	0.0225 (12)	0.0204 (11)	0.0014 (9)	−0.0045 (10)	−0.0001 (9)
N12	0.0272 (13)	0.0221 (12)	0.0173 (11)	0.0007 (9)	−0.0050 (10)	−0.0002 (9)
C1	0.0366 (18)	0.0272 (14)	0.0298 (16)	−0.0052 (12)	−0.0055 (13)	0.0071 (12)
C2	0.0235 (14)	0.0226 (13)	0.0255 (14)	−0.0034 (11)	0.0007 (11)	−0.0016 (11)
C3	0.0331 (17)	0.0247 (13)	0.0283 (15)	−0.0062 (12)	−0.0037 (13)	−0.0027 (11)
C4	0.0255 (16)	0.0290 (15)	0.0226 (16)	−0.0034 (12)	−0.0042 (13)	−0.0040 (12)
C5	0.0339 (17)	0.0418 (19)	0.042 (2)	−0.0102 (14)	−0.0151 (15)	0.0098 (15)
C6	0.0231 (15)	0.0357 (16)	0.0415 (18)	−0.0058 (13)	−0.0009 (13)	0.0017 (14)
C7	0.0229 (15)	0.0250 (14)	0.0332 (16)	−0.0020 (11)	0.0046 (12)	0.0037 (11)
C8	0.0266 (15)	0.0339 (16)	0.0310 (16)	0.0042 (12)	0.0083 (13)	0.0055 (13)
C9	0.0333 (15)	0.0273 (14)	0.0169 (12)	0.0063 (14)	0.0063 (11)	0.0015 (12)
C10	0.0403 (19)	0.0402 (17)	0.0234 (14)	0.0074 (14)	0.0048 (13)	−0.0015 (12)
C11	0.0305 (16)	0.0308 (16)	0.0233 (15)	0.0034 (12)	0.0023 (12)	−0.0029 (11)
C12	0.0189 (13)	0.0273 (14)	0.0204 (14)	0.0015 (11)	−0.0026 (11)	−0.0042 (10)
C13	0.0231 (15)	0.0252 (14)	0.0229 (14)	0.0029 (11)	−0.0006 (12)	−0.0010 (11)
C14	0.0188 (13)	0.0244 (14)	0.0216 (14)	−0.0004 (11)	−0.0024 (11)	0.0023 (11)
C15	0.052 (2)	0.0308 (16)	0.0255 (16)	0.0097 (14)	0.0038 (15)	0.0083 (13)
C16	0.0324 (17)	0.0276 (15)	0.0235 (15)	0.0049 (13)	0.0002 (13)	−0.0026 (11)
C17	0.0168 (13)	0.0233 (13)	0.0236 (14)	−0.0028 (10)	−0.0019 (11)	−0.0023 (10)
C18	0.0221 (15)	0.0201 (14)	0.0266 (15)	0.0012 (11)	−0.0007 (12)	0.0021 (11)
C19	0.0240 (14)	0.0230 (14)	0.0230 (15)	−0.0009 (11)	−0.0033 (12)	0.0031 (11)
C20	0.0400 (19)	0.0314 (15)	0.0238 (15)	0.0012 (13)	−0.0009 (13)	0.0043 (12)
C21	0.0283 (17)	0.0371 (17)	0.0424 (19)	−0.0086 (13)	−0.0004 (14)	0.0051 (14)
C22	0.0238 (15)	0.0254 (15)	0.0281 (15)	−0.0037 (11)	0.0011 (12)	0.0061 (11)
C23	0.0272 (16)	0.0320 (16)	0.0309 (16)	0.0034 (12)	0.0096 (13)	0.0050 (12)
C24	0.0266 (14)	0.0271 (14)	0.0246 (13)	0.0051 (13)	0.0065 (11)	0.0029 (13)
C25	0.0431 (19)	0.0468 (18)	0.0192 (14)	0.0087 (14)	0.0062 (13)	−0.0039 (12)
C26	0.0345 (18)	0.0245 (14)	0.0347 (17)	−0.0057 (12)	−0.0101 (14)	0.0040 (13)
C27	0.0281 (15)	0.0196 (13)	0.0223 (14)	−0.0007 (11)	−0.0039 (12)	−0.0005 (11)
C28	0.0269 (15)	0.0279 (14)	0.0315 (16)	−0.0100 (12)	−0.0058 (13)	0.0030 (12)
C29	0.0264 (16)	0.0282 (15)	0.0275 (17)	−0.0064 (12)	−0.0077 (13)	0.0013 (12)
C30	0.050 (2)	0.0425 (19)	0.039 (2)	−0.0139 (16)	−0.0255 (17)	0.0067 (15)
C31	0.0240 (13)	0.0274 (14)	0.0146 (11)	0.0008 (12)	−0.0024 (10)	−0.0003 (11)
C32	0.0196 (12)	0.0221 (13)	0.0200 (12)	−0.0007 (12)	0.0003 (10)	−0.0009 (11)
S1A	0.0242 (5)	0.0246 (4)	0.0221 (4)	−0.0043 (4)	0.0023 (3)	−0.0031 (3)
O1A	0.022 (2)	0.0380 (11)	0.043 (2)	−0.0014 (18)	0.0119 (15)	−0.0013 (13)
O2A	0.036 (2)	0.035 (2)	0.0327 (14)	0.0007 (14)	0.0000 (13)	0.0048 (15)
O3A	0.0367 (12)	0.0206 (17)	0.028 (3)	−0.0030 (14)	0.0035 (18)	−0.0111 (14)
C1A	0.032 (3)	0.027 (2)	0.036 (3)	−0.006 (2)	0.0129 (19)	0.000 (2)
F1A	0.0198 (18)	0.0387 (19)	0.0514 (13)	0.0018 (12)	−0.0010 (15)	−0.0025 (12)
F2A	0.0480 (14)	0.057 (2)	0.0211 (19)	−0.0175 (14)	−0.0004 (16)	0.0044 (14)
F3A	0.0349 (18)	0.0266 (11)	0.039 (3)	−0.0051 (11)	−0.0016 (15)	−0.0125 (11)
S1A'	0.0242 (5)	0.0246 (4)	0.0221 (4)	−0.0043 (4)	0.0023 (3)	−0.0031 (3)
O1A'	0.0198 (18)	0.0387 (19)	0.0514 (13)	0.0018 (12)	−0.0010 (15)	−0.0025 (12)
O2A'	0.0480 (14)	0.057 (2)	0.0211 (19)	−0.0175 (14)	−0.0004 (16)	0.0044 (14)
O3A'	0.0349 (18)	0.0266 (11)	0.039 (3)	−0.0051 (11)	−0.0016 (15)	−0.0125 (11)
C1A'	0.032 (3)	0.027 (2)	0.036 (3)	−0.006 (2)	0.0129 (19)	0.000 (2)
F1A'	0.022 (2)	0.0380 (11)	0.043 (2)	−0.0014 (18)	0.0119 (15)	−0.0013 (13)
F2A'	0.036 (2)	0.035 (2)	0.0327 (14)	0.0007 (14)	0.0000 (13)	0.0048 (15)
F3A'	0.0367 (12)	0.0206 (17)	0.028 (3)	−0.0030 (14)	0.0035 (18)	−0.0111 (14)

### Geometric parameters (Å, º)

**Table:**

Na1—N11	2.427 (3)	C12—C13	1.404 (4)
Na1—N1	2.445 (3)	C13—C14	1.375 (4)
Na1—N9	2.459 (3)	C13—H13	0.9500
Na1—N3	2.463 (3)	C14—C15	1.492 (4)
Na1—N7	2.501 (3)	C15—H15A	0.9800
Na1—N5	2.507 (3)	C15—H15B	0.9800
N1—C2	1.334 (4)	C15—H15C	0.9800
N1—N2	1.369 (3)	C16—C17	1.492 (4)
N2—C4	1.366 (4)	C16—H16A	0.9800
N2—C31	1.447 (4)	C16—H16B	0.9800
N3—C7	1.333 (4)	C16—H16C	0.9800
N3—N4	1.364 (3)	C17—C18	1.411 (4)
N4—C9	1.372 (3)	C18—C19	1.359 (4)
N4—C31	1.437 (3)	C18—H18	0.9500
N5—C12	1.331 (3)	C19—C20	1.491 (4)
N5—N6	1.375 (3)	C20—H20A	0.9800
N6—C14	1.360 (3)	C20—H20B	0.9800
N6—C31	1.450 (3)	C20—H20C	0.9800
N7—C17	1.335 (3)	C21—C22	1.495 (4)
N7—N8	1.368 (3)	C21—H21A	0.9800
N8—C19	1.376 (3)	C21—H21B	0.9800
N8—C32	1.450 (3)	C21—H21C	0.9800
N9—C22	1.327 (4)	C22—C23	1.410 (4)
N9—N10	1.374 (3)	C23—C24	1.372 (4)
N10—C24	1.359 (3)	C23—H23	0.9500
N10—C32	1.449 (3)	C24—C25	1.493 (4)
N11—C27	1.324 (4)	C25—H25A	0.9800
N11—N12	1.377 (3)	C25—H25B	0.9800
N12—C29	1.364 (4)	C25—H25C	0.9800
N12—C32	1.446 (4)	C26—C27	1.492 (4)
C1—C2	1.501 (4)	C26—H26A	0.9800
C1—H1A	0.9800	C26—H26B	0.9800
C1—H1B	0.9800	C26—H26C	0.9800
C1—H1C	0.9800	C27—C28	1.408 (4)
C2—C3	1.407 (4)	C28—C29	1.371 (4)
C3—C4	1.360 (4)	C28—H28	0.9500
C3—H3	0.9500	C29—C30	1.498 (4)
C4—C5	1.487 (4)	C30—H30A	0.9800
C5—H5A	0.9800	C30—H30B	0.9800
C5—H5B	0.9800	C30—H30C	0.9800
C5—H5C	0.9800	C31—H31	1.0000
C6—C7	1.489 (4)	C32—H32	1.0000
C6—H6A	0.9800	S1A—O2A	1.429 (5)
C6—H6B	0.9800	S1A—O1A	1.455 (4)
C6—H6C	0.9800	S1A—O3A	1.464 (6)
C7—C8	1.415 (4)	S1A—C1A	1.816 (7)
C8—C9	1.367 (4)	C1A—F1A	1.330 (9)
C8—H8	0.9500	C1A—F2A	1.334 (9)
C9—C10	1.487 (4)	C1A—F3A	1.344 (10)
C10—H10A	0.9800	S1A'—O2A'	1.424 (16)
C10—H10B	0.9800	S1A'—O1A'	1.459 (15)
C10—H10C	0.9800	S1A'—O3A'	1.462 (18)
C11—C12	1.496 (4)	S1A'—C1A'	1.799 (14)
C11—H11A	0.9800	C1A'—F1A'	1.336 (18)
C11—H11B	0.9800	C1A'—F3A'	1.364 (19)
C11—H11C	0.9800	C1A'—F2A'	1.376 (19)
			
N11—Na1—N1	179.48 (12)	C13—C14—C15	130.9 (3)
N11—Na1—N9	75.50 (9)	C14—C15—H15A	109.5
N1—Na1—N9	104.97 (9)	C14—C15—H15B	109.5
N11—Na1—N3	104.81 (9)	H15A—C15—H15B	109.5
N1—Na1—N3	74.71 (8)	C14—C15—H15C	109.5
N9—Na1—N3	179.54 (12)	H15A—C15—H15C	109.5
N11—Na1—N7	76.63 (8)	H15B—C15—H15C	109.5
N1—Na1—N7	103.24 (9)	C17—C16—H16A	109.5
N9—Na1—N7	79.31 (9)	C17—C16—H16B	109.5
N3—Na1—N7	100.42 (9)	H16A—C16—H16B	109.5
N11—Na1—N5	103.28 (9)	C17—C16—H16C	109.5
N1—Na1—N5	76.84 (8)	H16A—C16—H16C	109.5
N9—Na1—N5	101.18 (9)	H16B—C16—H16C	109.5
N3—Na1—N5	79.09 (8)	N7—C17—C18	111.0 (2)
N7—Na1—N5	179.47 (12)	N7—C17—C16	120.6 (2)
C2—N1—N2	104.4 (2)	C18—C17—C16	128.5 (2)
C2—N1—Na1	124.21 (19)	C19—C18—C17	106.8 (3)
N2—N1—Na1	114.06 (16)	C19—C18—H18	126.6
C4—N2—N1	112.2 (2)	C17—C18—H18	126.6
C4—N2—C31	127.0 (2)	C18—C19—N8	105.5 (3)
N1—N2—C31	120.3 (2)	C18—C19—C20	132.1 (3)
C7—N3—N4	105.0 (2)	N8—C19—C20	122.4 (3)
C7—N3—Na1	130.29 (19)	C19—C20—H20A	109.5
N4—N3—Na1	116.44 (17)	C19—C20—H20B	109.5
N3—N4—C9	112.4 (2)	H20A—C20—H20B	109.5
N3—N4—C31	120.4 (2)	C19—C20—H20C	109.5
C9—N4—C31	127.0 (2)	H20A—C20—H20C	109.5
C12—N5—N6	104.2 (2)	H20B—C20—H20C	109.5
C12—N5—Na1	122.83 (17)	C22—C21—H21A	109.5
N6—N5—Na1	111.19 (16)	C22—C21—H21B	109.5
C14—N6—N5	112.1 (2)	H21A—C21—H21B	109.5
C14—N6—C31	125.9 (2)	C22—C21—H21C	109.5
N5—N6—C31	120.9 (2)	H21A—C21—H21C	109.5
C17—N7—N8	104.2 (2)	H21B—C21—H21C	109.5
C17—N7—Na1	121.23 (17)	N9—C22—C23	111.5 (2)
N8—N7—Na1	111.07 (17)	N9—C22—C21	119.9 (3)
N7—N8—C19	112.5 (2)	C23—C22—C21	128.6 (3)
N7—N8—C32	120.57 (19)	C24—C23—C22	106.1 (3)
C19—N8—C32	126.0 (2)	C24—C23—H23	126.9
C22—N9—N10	104.0 (2)	C22—C23—H23	126.9
C22—N9—Na1	130.30 (19)	N10—C24—C23	105.6 (3)
N10—N9—Na1	115.87 (16)	N10—C24—C25	122.4 (3)
C24—N10—N9	112.8 (2)	C23—C24—C25	132.0 (3)
C24—N10—C32	127.1 (2)	C24—C25—H25A	109.5
N9—N10—C32	119.9 (2)	C24—C25—H25B	109.5
C27—N11—N12	104.8 (2)	H25A—C25—H25B	109.5
C27—N11—Na1	123.79 (19)	C24—C25—H25C	109.5
N12—N11—Na1	114.28 (16)	H25A—C25—H25C	109.5
C29—N12—N11	111.9 (2)	H25B—C25—H25C	109.5
C29—N12—C32	126.8 (2)	C27—C26—H26A	109.5
N11—N12—C32	120.6 (2)	C27—C26—H26B	109.5
C2—C1—H1A	109.5	H26A—C26—H26B	109.5
C2—C1—H1B	109.5	C27—C26—H26C	109.5
H1A—C1—H1B	109.5	H26A—C26—H26C	109.5
C2—C1—H1C	109.5	H26B—C26—H26C	109.5
H1A—C1—H1C	109.5	N11—C27—C28	111.1 (2)
H1B—C1—H1C	109.5	N11—C27—C26	120.5 (2)
N1—C2—C3	110.9 (3)	C28—C27—C26	128.3 (3)
N1—C2—C1	119.9 (2)	C29—C28—C27	106.3 (3)
C3—C2—C1	129.2 (3)	C29—C28—H28	126.9
C4—C3—C2	106.5 (3)	C27—C28—H28	126.9
C4—C3—H3	126.7	N12—C29—C28	105.9 (2)
C2—C3—H3	126.7	N12—C29—C30	122.5 (3)
C3—C4—N2	105.9 (2)	C28—C29—C30	131.5 (3)
C3—C4—C5	131.1 (3)	C29—C30—H30A	109.5
N2—C4—C5	122.9 (3)	C29—C30—H30B	109.5
C4—C5—H5A	109.5	H30A—C30—H30B	109.5
C4—C5—H5B	109.5	C29—C30—H30C	109.5
H5A—C5—H5B	109.5	H30A—C30—H30C	109.5
C4—C5—H5C	109.5	H30B—C30—H30C	109.5
H5A—C5—H5C	109.5	N4—C31—N2	112.3 (2)
H5B—C5—H5C	109.5	N4—C31—N6	110.8 (2)
C7—C6—H6A	109.5	N2—C31—N6	111.4 (2)
C7—C6—H6B	109.5	N4—C31—H31	107.3
H6A—C6—H6B	109.5	N2—C31—H31	107.3
C7—C6—H6C	109.5	N6—C31—H31	107.3
H6A—C6—H6C	109.5	N12—C32—N10	111.4 (2)
H6B—C6—H6C	109.5	N12—C32—N8	111.8 (2)
N3—C7—C8	110.4 (3)	N10—C32—N8	110.9 (2)
N3—C7—C6	120.4 (3)	N12—C32—H32	107.5
C8—C7—C6	129.2 (3)	N10—C32—H32	107.5
C9—C8—C7	106.7 (3)	N8—C32—H32	107.5
C9—C8—H8	126.6	O2A—S1A—O1A	114.7 (3)
C7—C8—H8	126.6	O2A—S1A—O3A	115.8 (3)
C8—C9—N4	105.5 (2)	O1A—S1A—O3A	115.2 (4)
C8—C9—C10	132.2 (3)	O2A—S1A—C1A	103.3 (4)
N4—C9—C10	122.3 (3)	O1A—S1A—C1A	102.7 (4)
C9—C10—H10A	109.5	O3A—S1A—C1A	102.3 (4)
C9—C10—H10B	109.5	F1A—C1A—F2A	107.5 (7)
H10A—C10—H10B	109.5	F1A—C1A—F3A	106.1 (7)
C9—C10—H10C	109.5	F2A—C1A—F3A	107.2 (7)
H10A—C10—H10C	109.5	F1A—C1A—S1A	112.4 (6)
H10B—C10—H10C	109.5	F2A—C1A—S1A	112.0 (6)
C12—C11—H11A	109.5	F3A—C1A—S1A	111.3 (5)
C12—C11—H11B	109.5	O2A'—S1A'—O1A'	114.1 (19)
H11A—C11—H11B	109.5	O2A'—S1A'—O3A'	111 (2)
C12—C11—H11C	109.5	O1A'—S1A'—O3A'	115 (2)
H11A—C11—H11C	109.5	O2A'—S1A'—C1A'	106.9 (15)
H11B—C11—H11C	109.5	O1A'—S1A'—C1A'	104.4 (12)
N5—C12—C13	111.7 (3)	O3A'—S1A'—C1A'	103.9 (14)
N5—C12—C11	119.9 (2)	F1A'—C1A'—F3A'	102.7 (19)
C13—C12—C11	128.4 (3)	F1A'—C1A'—F2A'	109.1 (16)
C14—C13—C12	105.7 (2)	F3A'—C1A'—F2A'	106.3 (18)
C14—C13—H13	127.2	F1A'—C1A'—S1A'	116.0 (16)
C12—C13—H13	127.2	F3A'—C1A'—S1A'	107.4 (15)
N6—C14—C13	106.2 (3)	F2A'—C1A'—S1A'	114.3 (13)
N6—C14—C15	122.8 (3)		
			
N11—Na1—N1—C2	149 (14)	Na1—N3—C7—C6	−33.8 (4)
N9—Na1—N1—C2	−7.8 (2)	N3—C7—C8—C9	0.6 (3)
N3—Na1—N1—C2	171.9 (2)	C6—C7—C8—C9	−179.9 (3)
N7—Na1—N1—C2	74.5 (2)	C7—C8—C9—N4	−0.3 (3)
N5—Na1—N1—C2	−106.0 (2)	C7—C8—C9—C10	179.0 (3)
N11—Na1—N1—N2	−81 (14)	N3—N4—C9—C8	0.0 (3)
N9—Na1—N1—N2	121.47 (18)	C31—N4—C9—C8	−174.7 (3)
N3—Na1—N1—N2	−58.88 (18)	N3—N4—C9—C10	−179.5 (3)
N7—Na1—N1—N2	−156.25 (17)	C31—N4—C9—C10	5.8 (4)
N5—Na1—N1—N2	23.21 (18)	N6—N5—C12—C13	−1.6 (3)
C2—N1—N2—C4	0.9 (3)	Na1—N5—C12—C13	125.8 (2)
Na1—N1—N2—C4	−137.74 (19)	N6—N5—C12—C11	178.0 (2)
C2—N1—N2—C31	173.5 (2)	Na1—N5—C12—C11	−54.7 (3)
Na1—N1—N2—C31	34.9 (3)	N5—C12—C13—C14	−0.1 (3)
N11—Na1—N3—C7	66.8 (2)	C11—C12—C13—C14	−179.6 (3)
N1—Na1—N3—C7	−113.0 (2)	N5—N6—C14—C13	−2.8 (3)
N9—Na1—N3—C7	−66 (15)	C31—N6—C14—C13	−171.2 (2)
N7—Na1—N3—C7	−12.0 (2)	N5—N6—C14—C15	176.9 (3)
N5—Na1—N3—C7	167.8 (2)	C31—N6—C14—C15	8.5 (4)
N11—Na1—N3—N4	−149.92 (18)	C12—C13—C14—N6	1.7 (3)
N1—Na1—N3—N4	30.28 (17)	C12—C13—C14—C15	−178.0 (3)
N9—Na1—N3—N4	77 (15)	N8—N7—C17—C18	1.6 (3)
N7—Na1—N3—N4	131.30 (18)	Na1—N7—C17—C18	−124.4 (2)
N5—Na1—N3—N4	−48.90 (18)	N8—N7—C17—C16	−178.3 (2)
C7—N3—N4—C9	0.4 (3)	Na1—N7—C17—C16	55.7 (3)
Na1—N3—N4—C9	−151.37 (19)	N7—C17—C18—C19	−0.4 (3)
C7—N3—N4—C31	175.5 (2)	C16—C17—C18—C19	179.5 (3)
Na1—N3—N4—C31	23.7 (3)	C17—C18—C19—N8	−1.0 (3)
N11—Na1—N5—C12	−2.8 (2)	C17—C18—C19—C20	177.4 (3)
N1—Na1—N5—C12	177.7 (2)	N7—N8—C19—C18	2.1 (3)
N9—Na1—N5—C12	74.8 (2)	C32—N8—C19—C18	170.7 (2)
N3—Na1—N5—C12	−105.6 (2)	N7—N8—C19—C20	−176.5 (2)
N7—Na1—N5—C12	−83 (13)	C32—N8—C19—C20	−7.9 (4)
N11—Na1—N5—N6	121.46 (16)	N10—N9—C22—C23	−0.4 (3)
N1—Na1—N5—N6	−58.03 (16)	Na1—N9—C22—C23	−143.7 (2)
N9—Na1—N5—N6	−160.99 (16)	N10—N9—C22—C21	−179.9 (2)
N3—Na1—N5—N6	18.63 (16)	Na1—N9—C22—C21	36.8 (4)
N7—Na1—N5—N6	41 (13)	N9—C22—C23—C24	0.5 (3)
C12—N5—N6—C14	2.7 (3)	C21—C22—C23—C24	179.9 (3)
Na1—N5—N6—C14	−131.50 (19)	N9—N10—C24—C23	0.1 (3)
C12—N5—N6—C31	171.8 (2)	C32—N10—C24—C23	175.4 (3)
Na1—N5—N6—C31	37.6 (3)	N9—N10—C24—C25	179.2 (3)
N11—Na1—N7—C17	−177.6 (2)	C32—N10—C24—C25	−5.5 (4)
N1—Na1—N7—C17	1.9 (2)	C22—C23—C24—N10	−0.3 (3)
N9—Na1—N7—C17	104.9 (2)	C22—C23—C24—C25	−179.3 (3)
N3—Na1—N7—C17	−74.7 (2)	N12—N11—C27—C28	1.6 (3)
N5—Na1—N7—C17	−97 (13)	Na1—N11—C27—C28	−131.9 (2)
N11—Na1—N7—N8	59.55 (16)	N12—N11—C27—C26	−179.0 (3)
N1—Na1—N7—N8	−120.96 (16)	Na1—N11—C27—C26	47.5 (4)
N9—Na1—N7—N8	−17.92 (16)	N11—C27—C28—C29	−1.0 (4)
N3—Na1—N7—N8	162.46 (16)	C26—C27—C28—C29	179.7 (3)
N5—Na1—N7—N8	140 (13)	N11—N12—C29—C28	1.1 (3)
C17—N7—N8—C19	−2.3 (3)	C32—N12—C29—C28	170.8 (3)
Na1—N7—N8—C19	129.9 (2)	N11—N12—C29—C30	−179.2 (3)
C17—N7—N8—C32	−171.6 (2)	C32—N12—C29—C30	−9.4 (5)
Na1—N7—N8—C32	−39.5 (3)	C27—C28—C29—N12	−0.1 (3)
N11—Na1—N9—C22	110.9 (2)	C27—C28—C29—C30	−179.8 (4)
N1—Na1—N9—C22	−69.3 (3)	N3—N4—C31—N2	−77.7 (3)
N3—Na1—N9—C22	−116 (15)	C9—N4—C31—N2	96.6 (3)
N7—Na1—N9—C22	−170.3 (2)	N3—N4—C31—N6	47.7 (3)
N5—Na1—N9—C22	9.9 (3)	C9—N4—C31—N6	−138.0 (3)
N11—Na1—N9—N10	−28.94 (18)	C4—N2—C31—N4	−148.6 (3)
N1—Na1—N9—N10	150.86 (18)	N1—N2—C31—N4	39.9 (3)
N3—Na1—N9—N10	104 (15)	C4—N2—C31—N6	86.4 (3)
N7—Na1—N9—N10	49.86 (18)	N1—N2—C31—N6	−85.1 (3)
N5—Na1—N9—N10	−129.94 (18)	C14—N6—C31—N4	77.0 (3)
C22—N9—N10—C24	0.2 (3)	N5—N6—C31—N4	−90.5 (3)
Na1—N9—N10—C24	149.8 (2)	C14—N6—C31—N2	−157.1 (2)
C22—N9—N10—C32	−175.4 (2)	N5—N6—C31—N2	35.4 (3)
Na1—N9—N10—C32	−25.9 (3)	C29—N12—C32—N10	149.7 (3)
N1—Na1—N11—C27	31 (14)	N11—N12—C32—N10	−41.3 (3)
N9—Na1—N11—C27	−172.2 (2)	C29—N12—C32—N8	−85.6 (3)
N3—Na1—N11—C27	8.1 (2)	N11—N12—C32—N8	83.3 (3)
N7—Na1—N11—C27	105.5 (2)	C24—N10—C32—N12	−95.4 (3)
N5—Na1—N11—C27	−73.9 (2)	N9—N10—C32—N12	79.6 (3)
N1—Na1—N11—N12	−99 (14)	C24—N10—C32—N8	139.4 (3)
N9—Na1—N11—N12	58.06 (19)	N9—N10—C32—N8	−45.6 (3)
N3—Na1—N11—N12	−121.59 (19)	N7—N8—C32—N12	−33.6 (3)
N7—Na1—N11—N12	−24.16 (18)	C19—N8—C32—N12	158.7 (2)
N5—Na1—N11—N12	156.38 (18)	N7—N8—C32—N10	91.4 (3)
C27—N11—N12—C29	−1.7 (3)	C19—N8—C32—N10	−76.4 (3)
Na1—N11—N12—C29	136.9 (2)	O2A—S1A—C1A—F1A	−62.6 (7)
C27—N11—N12—C32	−172.2 (2)	O1A—S1A—C1A—F1A	177.8 (7)
Na1—N11—N12—C32	−33.6 (3)	O3A—S1A—C1A—F1A	58.1 (7)
N2—N1—C2—C3	−0.6 (3)	O2A—S1A—C1A—F2A	176.2 (7)
Na1—N1—C2—C3	132.5 (2)	O1A—S1A—C1A—F2A	56.6 (7)
N2—N1—C2—C1	179.5 (3)	O3A—S1A—C1A—F2A	−63.1 (7)
Na1—N1—C2—C1	−47.4 (3)	O2A—S1A—C1A—F3A	56.3 (7)
N1—C2—C3—C4	0.1 (4)	O1A—S1A—C1A—F3A	−63.3 (7)
C1—C2—C3—C4	180.0 (3)	O3A—S1A—C1A—F3A	177.0 (8)
C2—C3—C4—N2	0.4 (3)	O2A'—S1A'—C1A'—F1A'	61 (2)
C2—C3—C4—C5	179.5 (3)	O1A'—S1A'—C1A'—F1A'	−177 (2)
N1—N2—C4—C3	−0.8 (3)	O3A'—S1A'—C1A'—F1A'	−57 (2)
C31—N2—C4—C3	−172.9 (3)	O2A'—S1A'—C1A'—F3A'	−53 (2)
N1—N2—C4—C5	−180.0 (3)	O1A'—S1A'—C1A'—F3A'	69 (2)
C31—N2—C4—C5	7.9 (5)	O3A'—S1A'—C1A'—F3A'	−171 (3)
N4—N3—C7—C8	−0.6 (3)	O2A'—S1A'—C1A'—F2A'	−170 (2)
Na1—N3—C7—C8	145.7 (2)	O1A'—S1A'—C1A'—F2A'	−49.1 (19)
N4—N3—C7—C6	179.9 (2)	O3A'—S1A'—C1A'—F2A'	72 (2)
